# Identification of *Paragonimus mexicanus* and *P. caliensis* in freshwater crabs from Costa Rica: evidence of zoonotic lung fluke diversity in Central America

**DOI:** 10.1371/journal.pntd.0013880

**Published:** 2026-01-13

**Authors:** Ingo S. Wehrtmann, Roderico Hernández-Chea, Célio Magalhães, Gaby Dolz, María José Zuniga Moya, Raquel Romero-Chaves, Fresia Villalobos-Rojas

**Affiliations:** 1 Unidad de Investigación Pesquera y Acuicultura (UNIP), Centro de Investigación en Ciencias del Mar y Limnología (CIMAR), Universidad de Costa Rica, San José, Costa Rica; 2 Museo de Zoología, Centro de Investigación en Biodiversidad y Ecología Tropical (CIBET), Universidad de Costa Rica, San José, Costa Rica; 3 Editorial Universitaria, Universidad del Valle de Guatemala, Guatemala City, Guatemala; 4 Maestría en Enfermedades Tropicales, Posgrado Regional en Ciencias Veterinarias (PCVET), Universidad Nacional, Barreal de Heredia, Heredia, Costa Rica; 5 Laboratório de Bioecologia e Sistemática de Crustáceos (LBSC), Departamento de Biologia, Faculdade de Filosofia, Ciências e Letras de Ribeirão Preto (FFCLRP), Universidade de São Paulo (USP). Av. Bandeirantes, Ribeirão Preto, São Paulo, Brazil; 6 Instituto Nacional de Pesquisas da Amazônia, Manaus, Brazil; 7 Laboratorio de Zoonosis y Entomología, Programa Medicina Poblacional, Escuela de Medicina Veterinaria, Universidad Nacional, Barreal de Heredia, Heredia, Costa Rica; 8 Programa de Doctorado en Ciencias, Sistema de Estudios de Posgrado, Universidad de Costa Rica, San José, Costa Rica; Stony Brook University, UNITED STATES OF AMERICA

## Abstract

*Paragonimus* spp. are foodborne trematodes with complex life cycles involving freshwater snails and decapod crustaceans. In Central America, *Paragonimus mexicanus* is a known zoonotic agent, whereas the public health significance of *P. caliensis* remains unclear. Between 2015 and 2016, we surveyed freshwater crabs across 51 sites in 15 river basins spanning three major climatic regions in Costa Rica. A total of 419 crabs (10 species) were examined for metacercariae; male crabs were identified morphologically, and infected females were identified to species by 16S rRNA and COI sequencing. Metacercariae were detected in six crab species, three of which represent new hosts of *Paragonimus*. The highest prevalence in male crabs occurred in *Ptychophallus uncinatus* (55.6%, 25/45). Molecular analyses confirmed the presence of two *Paragonimus* species in crabs: *P. mexicanus* and *P. caliensis*. A binomial GLM showed that males had higher odds of infection than females (p = 0.0059). Most infections occurred in areas along the Caribbean coast and in the Northern Region, which drains into Lake Nicaragua and Río San Juan. A binomial GLM revealed that the probability of *Paragonimus* infection varied significantly across climatic regions. Crabs from the Caribbean slope had the highest odds of infection, followed by the Northern Region. In contrast, crabs from the Pacific slope were significantly less likely to be infected. These patterns support the existence of geographic clusters of transmission within Costa Rica. We provide the first species-level, molecular confirmation of infected female crabs in Costa Rica and identify three freshwater crab species as newly recognized second intermediate hosts. These findings expand the known host range and distribution of *Paragonimus* in Costa Rica and highlight the need for public health education about the risks of consuming undercooked freshwater crabs.

## Author summary

Paragonimiasis is a foodborne parasitic infection caused by lung flukes of the genus *Paragonimus*, typically acquired through the consumption of raw or undercooked freshwater crabs harboring infective metacercariae. Although *Paragonimus* spp. have been reported in various crab species across Latin America, their distribution and host range in Costa Rica remain poorly understood. To address this gap, between 2015 and 2016, we collected a total of 419 freshwater crabs from 51 sites across 24 river basins in Costa Rica. Crab specimens were identified morphologically and examined for *Paragonimus* metacercariae using both microscopic and molecular techniques. Infection was detected in six crab species, three of which represent new hosts for *Paragonimus*. Male *Ptychophallus uncinatus* showed the highest infection prevalence (55.6%). Male crabs had significantly higher odds of infection than females (p = 0.0059), with infections most frequently detected in river basins draining into the Caribbean Sea and Lake Nicaragua. Molecular analyses identified two *Paragonimus* species: *Paragonimus mexicanus*, a known zoonotic agent, and *P. caliensis*, whose pathogenicity to humans remains uncertain. Notably, this study provides the first molecular identification of infected female freshwater crab hosts in the region. Our findings demonstrate that *Paragonimus* spp. in Costa Rica have a broader geographic and host range than previously recognized, underscoring the need for greater public health awareness regarding the consumption of freshwater crabs and for continued surveillance and research on the transmission dynamics of this neglected zoonotic parasite.

## Introduction

True freshwater crabs (Decapoda: Brachyura) act as second intermediate hosts of the lung flukes *Paragonimus* (Platyhelminthes), which are the etiological agents of paragonimiasis [1–3]. Human paragonimiasis is an important foodborne zoonotic disease; humans become infected by consuming raw or not fully cooked freshwater crabs containing metacercariae [2,4,5]. This parasitic infection is present in Asia, Africa, and the Americas, and according to the World Health Organization, there are at least 23 million people infected, with an estimated 293 million people in tropical and subtropical areas at risk of this disease [6,7]. However, the prevalence of paragonimiasis is very difficult to determine due to diagnostic problems (nonspecific symptoms, commonly confused with tuberculosis) and the lack of readily available laboratory diagnostic techniques [8].

Two species of lung flukes have been reported from Central America, *Paragonimus mexicanus* and *P. caliensis* [9–11]. However, recent molecular work suggests that *P. mexicanus* may represent a species complex with cryptic diversity across Central America [12], highlighting the possibility that more than two *Paragonimus* species circulate in the region. *Paragonimus mexicanus* has been detected in wild and domestic carnivorous mammals such as felines, foxes, raccoons, mustelids, and marsupials (opossums), and humans may also act as definitive hosts [13–17]. Adult parasites are commonly found in the lungs of definitive hosts, and the eggs are shed in sputum or feces [18], which develop into miracidia in freshwater bodies and penetrate the first intermediate hosts, small snails (in Central America, species of freshwater snails of the genus *Aroapyrgus*) [5]. The miracidia transform into sporocysts within the snails, which then give rise to rediae through asexual reproduction; in turn, rediae produce cercariae, which leave the snail and penetrate the second intermediate host, a freshwater crab (in Central America: pseudothelphusid crabs) [19]. Subsequently, the metacercariae (infective larvae) develop within the soft tissues of the crab (gills, heart, hepatopancreas, and muscles) [5,15]. The cycle is completed when definitive hosts ingest freshwater crabs infected with metacercariae [5]. *Paragonimus caliensis* has been detected so far only in two pseudothelphusid crab species, *Potamocarcinus magnus* and *Ptychophallus tristani* [9], whereas *P. mexicanus* has been previously reported only in *Ptychophallus tristani* [20]. To date, no confirmed human infections with *P. caliensis* have been reported in Costa Rica or elsewhere in Central America, and its zoonotic significance remains uncertain.

Roughly 30 cases of paragonimiasis have been officially reported from Costa Rica [11,17]. A study conducted in Talamanca, southern Costa Rica [17], revealed that 23% of schoolchildren (n = 100) were previously exposed to *P. mexicanus* infection, showing a high seroprevalence of this neglected tropical disease in children of this locality.

Rodríguez & Magalhães [19] listed 22 freshwater crab species from eight genera of the family Pseudothelphusidae as reported hosts for *Paragonimus* spp. in Latin America. The crab genera *Ptychophallus*, *Pseudothelphusa*, and *Hypolobocera* contain seven, five, and five species, respectively, the highest number of host species for lung flukes [19]. In a more recent compilation, Wehrtmann et al. [21] listed 15 pseudothelphusid crab species as hosts of *Paragonimus* spp. for Central America and Mexico. As pointed out by Rodríguez & Magalhães [19], all freshwater crab species reported as lung fluke hosts are relatively large species, and this fact may be associated with the human preference for large crabs, rather than the immunity of smaller freshwater crab species to these parasites. Additional studies are needed to better understand the role of freshwater crabs as second intermediate hosts in the transmission of *Paragonimus* spp. [19].

Our knowledge about freshwater crabs as hosts of lung flukes in Costa Rica is rather limited. *Ptychophallus tristani* and *Potamocarcinus magnus* have been mentioned by several authors as the host species of *Paragonimus* spp. [15,17,20,22,23], while *Ptychophallus costaricensis* and *Pseudothelphusa americana* (a species known from Mexico) were reported as hosts by Brenes et al. [10]. Probably the most detailed study conducted in Costa Rica on *Paragonimus* transmission reported an 88.5% infection rate with *P. mexicanus* metacercariae among 182 *Ptychophallus tristani* crabs analyzed [20].

A previous study in Costa Rica by Hernández-Chea et al. [11] focused on the molecular characterization of *Paragonimus* species, confirming *P. caliensis* as distinct from *P. mexicanus*. However, this study identified infected crabs only at the genus level (*Ptychophallus*, *Achlidon, Potamocarcinus*, and *Allacanthos*), without species-level resolution of the hosts. Building on this foundation, our study broadens the scope by conducting extensive sampling across three climatic regions, applying molecular tools to identify also infected female crabs at the species level, and analyzing host- and region-specific patterns of infection. This approach offers new insights into the role of freshwater crabs in the transmission of *Paragonimus* in Central America and provides valuable information to support public health awareness and control programs.

This study aimed to clarify the role of freshwater crabs in the transmission of *Paragonimus* in Costa Rica by providing species-level identification of infected crabs (including females identified by molecular techniques), expanding confirmed host records, and analyzing infection patterns across major climatic regions.

## Methods

### Collection and morphological identification of crab species

**Fig 1:**
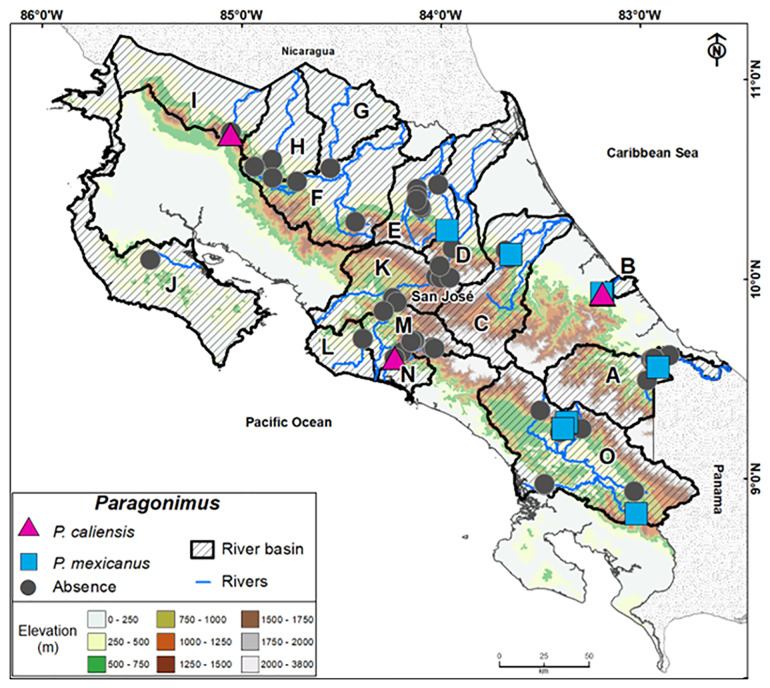
Sampling sites of freshwater crabs in Costa Rica across three major climatic regions (Caribbean, Pacific, and Northern Region). The rivers of the Northern Region drain into Lake Nicaragua, although only the Costa Rican portion was surveyed during 2015 and 2016 in this study. River basins **(A–O)**, elevation **(m)**, and occurrence of *Paragonimus* spp. metacercariae are shown. Squares indicate *P. mexicanus*, triangles *P. caliensis*, and gray circles indicate the absence of *Paragonimus*. Map prepared by the authors; country borders were obtained from GeoBoundaries (https://www.geoboundaries.org).

Costa Rica covers an area of 51,100 km^2^, and its hydrographic system is divided into 34 basins draining into three major climatic regions: the San Juan River biological corridor, the Caribbean Sea, and the Pacific Ocean [24]. Sampling was conducted between March 2015 and August 2016 at 51 locations distributed across 15 river basins (Table 1 and Fig 1). Crabs were collected manually during daylight by lifting rocks in or near small streams and other water bodies. Each specimen was placed in an individual plastic bag, stored on ice in a cooler at 4 °C, and then transported to the laboratory. Altitude and coordinates of each site were recorded using a GPS unit (Garmin Foretrex 401, Olathe, KS).

**Table 1:** Climatic regions and river basins of Costa Rica, with the number of sampling sites and coding used for mapping.

Climatic regions	Map Code	River Basin	# Sampling Sites
Caribbean	A	Río Sixaola	7
Caribbean	B	Río Moín	3
Caribbean	C	Río Reventazón	3
Caribbean	D	Río Chirripó	2
Northern Region	E	Río Sarapiquí	5
Northern Region	F	Río San Carlos	4
Northern Region	G	Río Pocosol	1
Northern Region	H	Río Frío	1
Northern Region	I	Río Zapote	2
Northern Region	J	Península de Nicoya	1
Pacific	K	Río Grande de Tárcoles	5
Pacific	L	Río Tusubres	1
Pacific	M	Río Parrita	6
Pacific	N	Río Damas	2
Pacific	O	Río Grande de Térraba	8

The first pair of male pleopods (gonopods) is the main morphological structure used for the taxonomic identification of pseudothelphusid crabs [25–29]. At least one gonopod was removed before dissection and preserved in 70% ethanol in a labeled vial, linked to each specimen’s collection data. Taxonomic identification was based on gonopod morphology following Magalhães et al. [30] and confirmed by comparison with voucher specimens deposited at the Crustacean Collection of the Museo de Zoología, Universidad de Costa Rica.

### Molecular identification of crabs

Because female pseudothelphusid crabs cannot be reliably identified to species level using somatic characters, infected females were prioritized for sequencing. Representative infected males from each locality, previously identified morphologically, were included solely to document which species was present at each site. Male and female sequences were not compared directly.

Genomic DNA was extracted from 200–500 mg of leg muscle tissue using a CTAB-based protocol [31], with modifications. Tissue was homogenized with a sterile pestle and incubated with preheated DNA lysis buffer (100 mM Tris-HCl pH 8.0, 1.4 M NaCl, 20 mM EDTA, 2% CTAB, 2% PVP, 2% β-mercaptoethanol) at 65 °C for 20 min. After chloroform–octanol extraction and isopropanol precipitation, the DNA pellet was washed with 70% ethanol, dried, resuspended in ultrapure water, quantified spectrophotometrically, and stored at −20 °C until use.

Fragments of the mitochondrial cytochrome oxidase I (COI; ~ 650 bp) and 16S rRNA (~500 bp) genes were amplified using primers COIA/COIF and 16SAR-L/16SBR-H, respectively [32]. PCR reactions (25 µL) contained 1X DreamTaq buffer, 0.2 mM dNTPs, 1 U Taq polymerase, 0.5 µM of each primer, and 100–200 ng DNA. Cycling conditions for 16S rRNA were: 95 °C for 3 min; 35 cycles of 95 °C for 30 s, 52 °C for 30 s, and 72 °C for 45 s; final extension at 72 °C for 5 min. For COI: same conditions except annealing at 48 °C.

PCR products were purified with Exonuclease I and FastAP (Thermo Scientific), sequenced bidirectionally with BigDye Terminator v3.1 on a 3130xl Genetic Analyzer (Applied Biosystems), and assembled in BIOEDIT v7.0.5 [33]. Consensus sequences from males and females were compared independently with reference sequences deposited in GenBank using the BLAST algorithm. Species identity was assigned based on concordance with the best BLAST matches and nucleotide similarity to validated reference sequences. No statistical or population-level comparisons between male and female sequences were conducted, as sequencing was performed solely for species confirmation. All newly generated sequences were deposited in GenBank. In localities where only a single crab species was confirmed morphologically (based on male specimens), sequencing one infected female was sufficient for molecular confirmation, as additional female sequences would have been redundant for species assignment.

### Crab dissection and identification of metacercariae

Crabs were euthanized via thermal shock (immersion in water at 2 °C for 10 min) [34]. Subsequently, the carapace, gills, hepatopancreas, heart, muscle tissue, chelae, and limbs were dissected in 0.9% saline and examined under a stereoscope (Nikon SM-5). Metacercariae were collected with a dropper, preserved in 0.9% saline or fixed in 70% ethanol, and examined under a light microscope (Nikon Eclipse E200, 10× and 40 × objectives). All crabs in which metacercariae were detected were included in the parasitological and molecular analyses. Metacercariae were initially examined morphologically, as *P. mexicanus* and *P. caliensis* can be distinguished based on size, cyst wall thickness, and internal structure [11]. Representative metacercariae from each infected crab were subsequently subjected to molecular confirmation following Hernández-Chea et al. [11].

### Molecular identification of *Paragonimus* spp.

Metacercariae isolated from infected crabs were subjected to DNA extraction and PCR to confirm parasite species identity. Genomic DNA was extracted from individual metacercariae using a CTAB-based protocol [11]. Fragments of the nuclear 28S rDNA and mitochondrial COI genes were amplified under the conditions described by Hernández-Chea et al. [11]. PCR products were purified with Exonuclease I and Alkaline Phosphatase (Thermo Scientific), sequenced bidirectionally with the same primers, and assembled in BIOEDIT v7.0.5 [33]. Consensus sequences were compared with GenBank references using BLAST and deposited under accession numbers KX289332–48 (28S rDNA) and KX344899–905, KX354390–92, KX379689–95 (COI).

### Statistical analysis

Prevalence estimates were calculated with 95% confidence intervals using the Wilson score method. Prevalence by species was calculated exclusively for males, since female crabs cannot be reliably identified to species morphologically. Differences in *Paragonimus* infection rates by crab species, sampling site, and sex were first evaluated using contingency tables and chi-square tests (with Fisher’s exact test when appropriate). To further assess group differences, we fitted generalized linear models (GLMs) with a binomial error distribution and logit link function. One model tested the effect of sex, and another evaluated climatic region effects (Caribbean, Northern Region, Pacific). Model outputs included estimated coefficients, standard errors, odds ratios, and 95% confidence intervals. All analyses were conducted in R version 4.3.1 [35], using the base stats package for GLMs, and in SPSS version 21.0 (IBM Corp.) for preliminary descriptive tests.

## Results

### Diversity of freshwater crab species

A total of 419 freshwater crabs were collected between March 2015 and August 2016 across 51 locations and 15 river basins in Costa Rica. The specimens included comprised 166 males and 253 females, including one ovigerous female, representing 10 species within four genera: *Achlidon* (1 sp.), *Allacanthos* (2 spp.), *Potamocarcinus* (3 spp.), and *Ptychophallus* (4 spp.).

The most abundant species were *Ptychophallus uncinatus* (45 males, 13 females) and *Pt. tristani* (51 males, 2 females), followed by *P. tumimanus* (15 males, 1 female), *Po. magnus* (14 males, 1 female), *Al. pittieri* (13 males), and *Al. yawi* (10 males, 2 females). The remaining species were rare, with ≤ 5 individuals collected. See Table 2 for the species list and infection status.

**Table 2:** Freshwater crab species collected in Costa Rica between March 2015 and August 2016. The table shows the number of infected individuals in relation to the number of collected individuals by sex.

Species	Infected/collected individuals (female)	Infected/collected individuals (male)
*Achlidon agrestis*	1/1	1/4
*Allacanthos pittieri*	–	0/13
*Allacanthos yawi*	2/2	2/10
*Potamocarcinus magnus*	1/1	4/14
*Potamocarcinus nicaraguensis*	–	0/4
*Potamocarcinus* sp.*	–	0/3
*Potamocarcinus montanus*	–	0/1
*Potamocarcinus richmondii*	–	0/4
*Ptychophallus tristani*	2/2	1/51
*Ptychophallus tumimanus*	1/1	0/15
*Ptychophallus uncinatus*	13/13	25/45
*Ptychophallus* sp.*	–	0/2
Not identified to species level	233	---
Total	20/253	33/166

* sp.= Not identified to species level

### Molecular identification of female crabs

Of the 253 female crabs examined, 20 were found to be infected with *Paragonimus* metacercariae. Of these, six infected females were further identified to species level using molecular techniques targeting the 16S rRNA and COI genes. The remaining infected females were not individually analyzed, as they were collected together with identified males in streams where only a single species was recorded [30]. We obtained sequences from six infected females (16S rDNA and COI for each) and sequences from six males (16S rRNAand COI), for a total of 24 sequences (see Table 3). Most infected female specimens were identified as *Ptychophallus uncinatus* (n = 13), followed by *P. tristani* (2), *Allacanthos yawi* (2), *Achlidon agrestis* (1), *Potamocarcinus magnus* (1), and *P. tumimanus* (1).

**Table 3:** Molecular identification of freshwater crab species infected with *Paragonimus* spp. in Costa Rica, based on 16S rRNA and COI gene sequences. Map codes refer to Table 1.

Species	Sex	16S identity (%/bp), Accession number	16S GenBank accession (this study)	COI identity (%/bp), Accession number	COI GenBank accession (this study)	River basin (map code)
***Achlidon agrestis***	Male	99.5 (409/411), MZ463238	PX354991	99.1 (547/552), MZ462213	PX358631	Río Chirripó (D)
	Female	98.8 (478/484), MZ463238	MN509183*	97.9 (558/579), MZ462213	PX358632	Río Reventazón (C)
***Allacanthos yawi***	Male	99.8 (506/507), MT868920	PX354990	98.2 (534/544), KY055670	PX358627	Río Damas (N)
	Female	100.0 (516/516), MT868920	MN509181*	97.8 (613/627), KY055670	PX358628	Río Grande de Térraba (O)
***Potamocarcinus magnus***	Male	99.6 (450/452), KU578886	PX354988	99.4 (494/497), KU578945	PX358629	Río Zapote (I)
	Female	99.4 (503/506), KU578886	PX354989	92.6 (500/540), KU578930	PX358630	Río Zapote (I)
***Ptychophallus uncinatus***	Male	99.2 (385/389), KU578852	PX354983	90.4 (496/549), MZ462214	PX358621	Río Moín (B)
	Female	99.4 (460/463), KU578853	PX354984	90.6 (529/524), KU578944	PX358622	Río Moín (B)
***Ptychophallus tristani***	Male	99.8 (433/434), MT868917	PX354986	98.7 (518/525), KU578944	PX358625	Río Damas (N)
	Female	99.8 (507/508), MT868917	PX354987	98.6 (636/645), KU578944	PX358626	Río Damas (N)
***Ptychophallus tumimanus***	Male	91.0 (387/425), OR116828	PX354985	98.3 (647/659), KU578941	PX358623	Río Zapote (I)
	Female	99.5 (420/422), MN509182	MN509182*	98.2 (539/549), KU578941	PX358624	Río Parrita (M)

Note: *Sequences that were previously deposited in GenBank by L. Barboza. We deposited the remaining 21 sequences (16S and COI) to complete the dataset avoiding redundancy. bp = base pairs.

Nucleotide identity values ranged from 98.76% to 100% for the 16S rRNA gene, and from 90.4% to 98.6% for the COI gene, when compared to reference sequences of the same crab species deposited in GenBank. New sequences generated in this study were deposited in GenBank (see Table 3).

#### Overall infection prevalence of freshwater crabs by *Paragonimus* spp.

Overall infection prevalence among the 419 crabs was 12.6% (95% CI: 9.6–16.1%). Male crabs showed a significantly higher prevalence (19.9%, 95% CI: 14.6–26.5; 33/166) than females (7.9%, 95% CI: 5.1–11.7; 20/253) (χ² test, P < 0.01). A generalized linear model (GLM) confirmed that male crabs had significantly higher odds of infection (OR = 2.36, 95% CI: 1.10–6.88, P = 0.0059). Two species of *Paragonimus* were identified in the infected crabs: *Paragonimus mexicanus*, which was predominant, and *P. caliensis*, which was less frequent and occurred with *P. mexicanus* in coinfections.

### Infection by crab species

Metacercariae were detected in six crab species and were absent in *Al. pittieri*, *Po. nicaraguensis*, *P. richmondii*, and *P. montanus*. Among morphologically identified males, *P. uncinatus* showed a prevalence of 55.6% (25/45). Additionally, 13 *Paragonimus*-positive females were identified, including one of them molecularly confirmed as *P. uncinatus*. Since only infected females were sequenced, prevalence by species could not be calculated for females.

### Climatic region-based infection patterns

Infection was unevenly distributed among the three climatic regions (Table 4). In the Caribbean Region, male crabs showed a prevalence of 43.1%, compared to 15.6% in the Northern Region and 3.9% in the Pacific Region. Female prevalence followed the same pattern, with the highest values in the Caribbean Region (22.5%) compared to the Northern Region (2.7%) and Pacific (3.2%).

**Table 4:** Prevalence of *Paragonimus* spp. infection in freshwater crabs by sex, climatic region, and river basin (March 2015 – August 2016). Values indicate the number of infected individuals per total examined, followed by prevalence (%) and 95% confidence intervals based on exact binomial calculations.

Climatic region	River Basin (Map code)	Infected Males/Total (%, 95% CI)	Infected Females/Total (%, 95% CI)
Caribbean	Río Sixaola (A)	0/12 (0.0%, 0.0–24.2)	1/18 (5.6%, 0.0–26.0)
Caribbean	Río Moín (B)	23/48 (47.9%, 34.5–61.7)	13/38 (34.2%, 21.2–50.1)
Caribbean	Río Reventazón (C)	0/4 (0.0%, 0.0–49.0)	1/3 (33.3%, 6.1–79.2)
Caribbean	Río Chirripó (D)	1/1 (100.0%, 20.7–100.0)	0/3 (0.0%, 0.0–56.1)
Northern Region	Río Sarapiquí (E)	1/4 (25.0%, 4.6–69.9)	0/7 (0.0%, 0.0–35.4)
Northern Region	Río San Carlos (F)	0/1 (0.0%, 0.0–79.3)	0/4 (0.0%, 0.0–49.0)
Northern Region	Río Pocosol (G)	0	0/1 (0.0%, 0.0–79.3)
Northern Region	Río Frío (H)	0/2 (0.0%, 0.0–65.8)	0/4 (0.0%, 0.0–49.0)
Northern Region	Río Zapote (I)	5/6 (83.3%, 43.6–97.0)	1/9 (11.1%, 2.0–43.5)
Northern Region	Península de Nicoya (J)	0	0/4 (0.0%, 0.0–49.0)
Pacific	Río Grande de Tárcoles (K)	0/9 (0.0%, 0.0–29.9)	0/11 (0.0%, 0.0–25.9)
Pacific	Río Tusubres (L)	0	0/4 (0.0%, 0.0–49.0)
Pacific	Río Parrita (M)	0/19 (0.0%, 0.0–16.8)	1/58 (1.7%, 0.3–9.1)
Pacific	Río Damas (N)	2/3 (66.7%, 20.8–93.9)	2/5 (40.0%, 11.8–76.9)
Pacific	Río Grande de Térraba (O)	1/57 (1.8%, 0.3–9.3)	1/84 (1.2%, 0.2–6.5)
Total		33/166 (19.9%, 14.5–26.6)	20/253 (7.9%, 5.2–11.9)

A binomial GLM confirmed that infection probability varied significantly among climatic regions. Crabs from the Caribbean had significantly higher odds of infection compared to those from the Pacific (OR = 4.81, 95% CI: 2.08–13.33, p < 0.001). Crabs from the Northern Region (Lake Nicaragua and Río San Juan) also showed increased odds compared to the Pacific (OR = 2.36, 95% CI: 1.10–6.88, p = 0.044). These results support the presence of spatial clustering of *Paragonimus* transmission risk (see Table 4 and Fig 1).

### Intensity, species identity, and distribution of metacercariae

A total of 53 crabs infected with *Paragonimus* metacercariae were examined. Morphological analyses following Hernández-Chea et al. [11] enabled the differentiation of *P. mexicanus* and *P. caliensis*, Coinfections were identified in individuals harboring metacercariae of both morphotypes and were subsequently confirmed by sequencing. Most crabs harbored 1–3 metacercariae, but one *P. uncinatus* female harbored 85 parasites. Only *Potamocarcinus magnus* (1 female, 22 metacercariae) and *P. uncinatus* (15 males, 3 females; 11–80 parasites) exhibited parasite intensities exceeding 10 metacercariae. The mean burden in *P. uncinatus* was 19.9 ± 22.86 SD. Other species, such as *Allacanthos yawi* (1 female, 2 metacercariae), had relatively low parasite loads.

The molecular identification of 53 metacercariae from infected crabs confirmed *P. mexicanus* as the predominant species, detected alone in 43 individuals and co-occurring with *P. caliensis* in three cases. *P. caliensis* alone was detected in seven crabs.

Coinfections were exclusively found in *P. uncinatus* from Río Moín (B), which also had the highest number of *P. mexicanus*-positive crabs (Table 4). *Paragonimus caliensis* was primarily found in *Potamocarcinus magnus* (Río Zapote, I), *P. tumimanus* (Río Parrita, M), and *P. tristani* (Río Damas, N) (Fig 1).

Table 5 provides updated records of pseudothelphusid crabs serving as *Paragonimus* hosts across the Neotropics, including findings from the present study.

**Table 5:** Published records of *Paragonimus* spp. infection in freshwater crabs (family Pseudothelphusidae) across Neotropics, including the results of the present study. Reported *Paragonimus* species are based on morphological or molecular identification as cited in the original references.

Host species	Country	*Paragonimus* species	Reference
**PSEUDOTHELPHUSIDAE**			
*Achlidon agrestis*	Costa Rica	*P. mexicanus*	Present study
*Allacanthos yawi*	Costa Rica	*P. mexicanus*	Present study
*Hypolobocera aequatorialis*	Ecuador	*P. mexicanus*	[8,19]
*H. bouvieri monticola*	Colombia	*Paragonimus* spp.	[19,36,37]
*H. bouvieri*	Colombia	*P. caliensis*	[38,39]
*H. chilensis*	Peru, Ecuador	*P. caliensis, P. amazonicum, P. mexicanus*	[8,19]
*H. emberara*	Colombia	*Paragonimus* spp.	[19]
*Lindacatalina gracilignatha*	Peru	*P. inca*	[19]
*Neostrengeria macropa*	Colombia	*Paragonimus* spp.	[40]
*Odontothelphusa maxillipes*	Mexico	*P. mexicanus*	[19]
*Potamocarcinus magnus*	Costa Rica	*P. caliensis, P. mexicanus*	[19] Present study
*Po. richmondi*	Panama, Costa Rica	*P. mexicanus*	[19]
*Pseudothelphusa belliana*	Mexico	*P. mexicanus*	[19]
*Ps.* aff. *seiferti*	Mexico	*P. mexicanus*	[41]
*Ps. dilatata*	Mexico, Costa Rica	*P. mexicanus*	[19]
*Ps. guerreroensis*	Mexico	*P. mexicanus*	[42]
*Ps. nayaritae*	Mexico	*P. mexicanus*	[19]
*Ps. propinqua*	Guatemala	*P. mexicanus*	[19]
*Ps. terrestris*	Mexico	*P. mexicanus*	[19]
*Ptychophallus coclensis*	Panama	*P. mexicanus*	[19]
*Pt. costaricensis*	Costa Rica	*P. mexicanus*	[19]
*Pt. exilipes*	Panama	*P. caliensis*	[19]
*Pt. tristani*	Costa Rica	*P. caliensis, P. mexicanus*	[19,20] Present study
*Pt. tumimanus*	Costa Rica	*P. mexicanus*	[19] Present study
*Pt. uncinatus*	Costa Rica	*P. caliensis, P. mexicanus*	Present study
*Raddaus boucourti*	Guatemala	*P. mexicanus*	[19]
*R. tuberculatus*	Mexico	*P. mexicanus*	[19,41]
*Rodriguezus garmani*	Venezuela	*P. mexicanus*	[19,43]
*Strengeria* sp.	Colombia	*P. caliensis*	[19,39]
*Strengeriana bolivarensis*	Colombia	*Paragonimus* sp.	[36]
*Sn. fuhrmanni*	Colombia	*Paragonimus* sp.	[36]
*Tehuana poglayenora*	Mexico	*P. mexicanus*	[41]

## Discussion

This study provides the most comprehensive assessment to date of *Paragonimus* infection in freshwater crabs from Costa Rica, integrating both morphological and molecular approaches. One of the major methodological contributions is the molecular identification of infected female crabs to the species level. As female pseudothelphusid crabs cannot be reliably distinguished based on external morphology [30], their role in the transmission cycle has often been overlooked. We were able to assign 20 infected females to six species (Table 2), using sequencing of 16S rRNA and COI genes of six individuals. In contrast, the other infected females were assigned to the only species collected in the stream, and where morphologically identified males were obtained together with these females in the same locality. To our knowledge, this is the first molecular characterization of infected female crabs not only in Costa Rica, and the results obtained highlight the value of molecular tools for resolving host–parasite associations and refining prevalence estimates [11,32].

In terms of host diversity, we examined 10 out of 15 freshwater crab species (66.7%) currently known from Costa Rica. Six species were found to harbor *Paragonimus* metacercariae. These findings expand the host spectrum by adding three new species as host species (*Achlidon agrestis*, *Allacanthos yawi*, and *Ptychophallus uncinatus*). This broad host range suggests that the diversity of susceptible pseudothelphusid hosts in Central America is greater than previously recognized.

Within this diverse host assemblage, clear sex-related patterns of infection emerged. Male crabs were more than twice as likely to be infected as females, as confirmed by both chi-square testing and a GLM (p = 0.0059, OR = 2.36). It is assumed that this disparity may be driven by behavioral or ecological differences between sexes, such as variation in mobility, habitat use, or dietary preferences. Similar male-biased infection patterns have been reported in pseudothelphusid crabs in Ecuador [34]. Further experimental studies will be necessary to confirm whether these differences reflect exposure risk, susceptibility, or both.

Beyond host-related patterns, molecular analyses confirmed the presence of two species of *Paragonimus*. *Paragonimus mexicanus* was the predominant species, found across multiple hosts and localities, while *P. caliensis* was detected only in *Po. magnus*, *P. tristani, P. tumimanus,* and *P. uncinatus*. The repeated detection of *P. caliensis*, though at lower frequencies, underscores the need to evaluate its potential zoonotic significance. However, to date, no confirmed human infections with *P. caliensis* have been reported in Costa Rica or elsewhere in Central America. Its presence in freshwater crabs highlights the need for further studies to assess its epidemiological role and potential risk to human health. In this context, our survey shows that *P. mexicanus* is broadly distributed across hosts and sites, whereas *P. caliensis* occurs only in a restricted set of crab species and always at low prevalence. This suggests that *P. caliensis* may have narrower host specificity or ecological requirements.

Our findings also contrast with Monge et al. [20], who reported 88.5% of *P. tristani* infected with *P. mexicanus*. In our study, *P. tristani* harbored only *P. mexicanus* (1/53) and *P. caliensis* (2/53) at very low prevalence. These discrepancies may reflect earlier misidentifications and underscores the importance of molecular confirmation when assessing host-parasite association.

In the context, it is noteworthy that although nucleotide identity values for the 16S rRNA gene were consistently high (≥ 98.7–100%), COI sequences in some cases showed lower identity values (down to ~90.4%) relative to GenBank references. These lower percentages likely reflect the limited availability and incongruence of COI sequences for Central American freshwater crabs in public databases, rather than uncertainty in species assignment [44]. Our identifications were further supported by congruent 16S rRNA results, as well as morphological confirmation of males, providing additional confidence. Nonetheless, the lack of comprehensive molecular reference data for pseudothelphusid crabs highlights the need for broader sequencing efforts in the region to strengthen molecular identifications and clarify phylogeographic patterns [45].

Our findings are consistent with a recent study suggesting that *P. mexicanus* represents a species complex with geographic structure [12]. This recognition of cryptic diversity may explain spatial heterogeneity in prevalence and underscores the need for multilocus or genomic studies in future surveys. Historical reports, such as Monge et al. [20], which documented high infection rates in *P. tristani*, may reflect site-specific transmission foci or misidentifications made before the availability of molecular tools.

Supporting this interpretation, our geographic analysis revealed strong spatial clustering of infection. Crabs from the Caribbean slope and the Northern Region showed a significantly higher prevalence compared to those from the Pacific slope. For example, the Río Moín basin exhibited infection rates exceeding 50% in male crabs, while infections were rare in Pacific sites. These findings align with earlier field observations of endemic foci in Limón and Talamanca [9] and with serological evidence of human exposure in the same regions [17]. In contrast, the low prevalence in Pacific drainages may reflect ecological or hydrological barriers that limit the distribution of the parasite or snail host (*Aroapyrgus* spp.) [46].

Although we did not record the distribution of the first intermediate snail host (*Aroapyrgus* spp.) at our sampling sites, its presence is likely to influence the spatial heterogeneity of infection. Previous work has shown that *A. costaricensis* can occur across a wide altitudinal range in Costa Rica, from near sea level to montane streams [47]. Considering the host specificity of *Paragonimus* in their snail hosts, the local absence or presence of these snails could explain why some sites within suitable altitudinal ranges showed no crab infections. In addition, anthropogenic factors such as deforestation, river basin connectivity, human settlements, and cultural practices related to crab consumption may further shape the spatial distribution of transmission foci, as suggested for Mesoamerica [12]. Future studies integrating snail surveys, ecological niche modeling, and analysis of socio-environmental variables will be crucial to evaluate whether the absence of infected crabs at some sites reflects the local absence of the snail host or broader ecological and human-driven barriers. Beyond spatial patterns of presence and absence, our data also revealed marked variation in infection density among host species.

Infection intensity varied widely among host species*. Ptychophallus uncinatus* exhibited the highest prevalence and parasite burdens, with up to 85 metacercariae in a single individual, indicating that this species may be a highly competent intermediate host. Other species, such as *Al. yawi* and *P. tumimanus*, showed much lower parasite loads, suggesting interspecific differences in host competence and transmission potential. Identifying such key host species is essential for understanding transmission dynamics and prioritizing surveillance. A broader comparison of infection rates and intensities across Neotropics, including our data and previously published studies, is summarized in S1 Table.

Comparable studies from Mexico, Guatemala, Colombia, Ecuador, Peru, and Venezuela have reported *Paragonimus* metacercariae in freshwater crabs, often focusing on single host species or limited geographic areas [9,19,20,23,37–43]. Previous regional compilations indicated that 22 pseudothelphusid crab species act as second intermediate hosts [19]. Our results not only confirm previous reports but also broaden the spectrum by documenting three new host species (*Achlidon agrestis*, *Allacanthos yawi*, and *Ptychophallus uncinatus*), with the added methodological advance of molecularly identifying infected females. This combination of broader sampling and molecular precision provides a clearer picture of host–parasite associations in Central America. Importantly, these expanded host associations have direct implications beyond ecology and systematics.

From a public health perspective, although only ~30 human cases of paragonimiasis have been officially reported in Costa Rica [11,17,43], our results suggest that the risk is broader and likely underestimated. The combination of widespread infected crabs confirmed zoonotic *Paragonimus* species, and evidence of human exposure calls for renewed health education campaigns, particularly in Caribbean and northern regions where infection rates were highest. Preventive efforts should focus on discouraging the consumption of raw or undercooked freshwater crabs. A recent community survey confirmed that 21% of respondents reported freshwater crab consumption in Costa Rica [48], underscoring the persistence of this risk behavior and the diagnostic challenges of paragonimiasis in the region.

Consistent with this, our results demonstrate that *Paragonimus* spp. infections in Costa Rica are concentrated in specific ecological contexts, particularly in the Caribbean and Northern Region, where higher prevalences were recorded and where freshwater crab consumption persists. Identifying key host species such as *Pt. uncinatus*, which harbored the highest infection intensities, highlights focal points of transmission risk. From a One Health perspective, these findings justify the implementation of targeted education campaigns in regions where infected crabs were collected, especially in rural and indigenous communities, as well as continued training of medical personnel to improve case recognition. Strengthening diagnostic capacity and culturally tailored health interventions is essential to reduce the risk of paragonimiasis, particularly in areas where the consumption of freshwater crabs remains common.

**Future directions**. While our study provides the most comprehensive survey to date of *Paragonimus* infection in freshwater crabs from Costa Rica, further steps are needed to fully understand the ecological drivers of transmission. Predictive distribution modeling approaches (e.g., MaxEnt) could be applied to integrate environmental variables, landscape features, and human settlement data, thereby identifying ecological determinants of infection risk. Similar approaches have already been used in Mesoamerica to demonstrate the influence of connectivity and human-modified landscapes on *Paragonimus* occurrence [12]. Such models would complement our findings by providing a predictive framework to guide targeted surveillance and control strategies in regions where infected freshwater crabs were collected.

Nevertheless, despite the extensive sampling, some limitations remain. Smaller or cryptic crab species may have been underrepresented, and we did not examine the first intermediate snail host or conduct experimental infections in definitive hosts. Future work should integrate ecological predictors of infection hotspots, multilocus or genomic markers for species delimitation, and serological surveys in at-risk human populations. These efforts will be crucial to fully elucidate transmission dynamics and to develop effective control strategies for paragonimiasis in Central America.

## Conclusion

This study provides the most comprehensive assessment of *Paragonimus* infection in freshwater crabs from Costa Rica, integrating morphological and molecular approaches to address long-standing taxonomic and epidemiological gaps. We identified six crab species as second intermediate hosts, including three newly reported host species, substantially expanding the known host range of *Paragonimus* in Central America. Molecular confirmation of infected female crabs represents a key methodological advance, enabling species-level resolution, previously unattainable for pseudothelphusid hosts. Infection patterns revealed significant sex bias and strong geographic structuring, with higher prevalence in the Caribbean and Northern Region. The co-occurrence of *P. mexicanus* and *P. caliensis* in multiple host species highlights ecological complexity within the transmission system and underscores the need for continued surveillance. Together, our results position freshwater crabs as critical components of the transmission cycle and provide an integrated framework to inform targeted public health interventions and future One Health-oriented research on paragonimiasis in Central America.

## Supporting information

S1 Table Comparison of published data, including the present study, on freshwater crab infections with *Paragonimus* spp. in the Neotropics. (DOCX)
