# Supplementary material for: Identification of Paragonimus mexicanus and P. caliensis in freshwater crabs from Costa Rica: evidence of zoonotic lung fluke diversity in Central America
Source: PLoS Negl Trop Dis. 2026 Jan 13;20(1):e0013880. doi: 10.1371/journal.pntd.0013880 (PMC12799009; doi:10.1371/journal.pntd.0013880)
Supplement: S1 Table — (DOCX) [file pntd.0013880.s001.docx]

**Wehrtmann et al.**

**Supplementary Table 1.** Comparison of published data, including the present study, on freshwater crab infections with *Paragonimus* spp. in the Neotropics.

| **Freshwater crab species** | **Country** | **Infection rate %*** | **# of ind. analyzed** | **Average # metacercariae /ind** | **Max # metacercariae /ind** | **Reference** |
| --- | --- | --- | --- | --- | --- | --- |
| *Achlidon agrestis* | Costa Rica | 40.0 | 5 | 1 | 1 | Present study |
| *Allacanthos yawi* | Costa Rica | 33.3 | 12 | 1.67 | 2 | Present study |
| *Eudaniela garmani* | Venezuela | 4.1; 18.0; 22.7 | 123; 634; 22 | NA | NA | [43] |
| *Hypolobocera aequatorialis* | Ecuador | 42.6; 68.5 | NA | NA | NA | [8] |
| *Hypolobocera bouvieri* | Colombia | 29.4 | 17 | NA | 58 | [39] |
| *Hypolobocera bouvieri* | Colombia | 55.5 | 27 | 6 | NA | [38] |
| *Hypolobocera chilensis* | Ecuador | 16.1 | NA | NA | NA | [8] |
| *Potamocarcinus magnus* | Costa Rica | 33.3 | 15 | 1.67 | 22 | Present study |
| *Potamocarcinus nicaraguensis* | Costa Rica | 0 | 4 | 0 | 0 | Present study |
| *Potamocarcinus richmondii* | Costa Rica | 0 | 4 | 0 | 0 | Present study |
| *Pseudothelphusa aff. seiferti* | Mexico | 40.0 | 10 | NA | NA | [41] |
| *Ptychophallus montanus* | Costa Rica | 0 | 1 | 0 | 0 | Present study |
| *Ptychophallus tristani* | Costa Rica | 88.5 | 182 | NA | NA | [20] |
| *Ptychophallus tristani* | Costa Rica | 5.7 | 53 | 1 | 1 | Present study |
| *Ptychophallus tumimanus* | Costa Rica | 6.3 | 16 | 1 | 1 | Present study |
| *Ptychophallus uncinatus* | Costa Rica | 65.5 | 58 | 19.9 | 85 | Present study |
| *Neostrengeria macropa* | Colombia | 17.2 | 29 | NA | NA | [40] |
| *Raddaus tuberculatus* | Mexico | 13.3 | 60 | NA | NA | [41] |
| *Strengeriana spp./Hypolobocera* sp. | Colombia | 80.8 | 52 | NA | NA | [36] |
| *Strengeria* sp. | Colombia | 20.0 | 10 | NA | 7 | [39] |
| *Tehuana guerreroensis* | Mexico | 20.8 | 120 | 1.9 | 6 | [42] |
| *Tehuana poglayenora* | Mexico | 14.5 | 48 | NA | NA | [41] |
